# Leukocytospermia does not negatively impact outcomes in in vitro fertilization cycles with intracytoplasmic sperm injection and preimplantation genetic testing for aneuploidy: findings from 5435 cycles

**DOI:** 10.1007/s10815-024-03085-x

**Published:** 2024-04-20

**Authors:** Pavan Gill, Nicolas Garrido Puchalt, Thomas Molinaro, Marie Werner, Emre Seli, James Hotaling, Philip Cheng

**Affiliations:** 1 IVI-RMA New Jersey, Basking Ridge, NJ USA; 2 IVI Foundation, Valencia, Spain; 3 School of Medicine Andrology and IVF Laboratories, University of Utah, Salt Lake City, UT USA

**Keywords:** Leukocytospermia, Embryological outcomes, Clinical outcomes, IVF with ICSI, PGT-A

## Abstract

**Purpose:**

To investigate whether leukocytospermia (defined as the presence of ≥ 1 × 10^6^ white blood cells/mL) affects clinical and embryologic outcomes in in vitro fertilization (IVF) cycles with intracytoplasmic sperm injection (ICSI) and preimplantation genetic testing for aneuploidy (PGT-A).

**Methods:**

This was a retrospective cohort study including 5425 cycles between January 2012 to December 2021 at a single large university-affiliated fertility clinic. The primary outcome was live birth rate (LBR).

**Results:**

The prevalence of leukocytospermia was 33.9% (*n* = 1843). Baseline characteristics including female age, BMI, AMH, Day 3 FSH, and male partner’s age were similar in cycles with and without leukocytospermia. The LBR after the first euploid embryo transfer was similar in those with and without leukocytospermia (62.3% vs. 63% *p* = 0.625). Secondary outcomes including clinical pregnancy rate (CPR), sustained implantation rate (SIR), fertilization (2PN) rate, blastulation rate, and aneuploidy rate were also evaluated. The CPR (73.3% vs 74.9%, *p* = 0.213) and SIR (64.6% vs. 66%, *p* = 0.305) were similar in both groups. The 2PN rate was also similar in both groups (85.7% vs. 85.8%, *p* = 0.791), as was the blastulation rate per 2PN (56.7% vs. 57.5%, *p* = 0.116). The aneuploidy rate was not significantly different between groups (25.7% vs 24.4%, *p* = 0.053). A generalized estimation equation with logistic regression demonstrated that the presence leukocytospermia did not influence the LBR (adjusted OR 0.878; 95% CI, 0.680–1.138).

**Conclusion:**

Leukocytospermia diagnosed just prior to an IVF cycle with PGT-A does not negatively impact clinical or embryologic outcomes.

## Introduction

Leukocytospermia, defined as the presence of ≥ 1 × 10^6^ white blood cells/mL of semen according to the 6th edition of the WHO Manual for the Laboratory Examination and Processing of Human Semen [1], is commonly identified in the semen analyses of men attending fertility clinics, with a prevalence of 10–40% [2, 3]. Given its relatively high occurrence amongst this population, it has been suggested that leukocytospermia may contribute to male factor infertility, even in the absence of inflammatory symptoms or an overt genital tract infection [3]. It has been associated with abnormal semen parameters, including decreased progressive motility and sperm concentration as well as adverse ART outcomes including decreased fertilization and embryo development rates despite the use of in vitro fertilization (IVF) and intracytoplasmic sperm injection (ICSI) [4]. One key postulated mechanism is related to the increased levels of reactive oxidative species (ROS) in the presence of leukocytospermia. This can damage spermatozoa, negatively impact fertilization capacity, and adversely influence semen parameters including sperm concentration and motility [5–7]. An increase in DNA fragmentation has also been linked to leukocytospermia [8]. This could theoretically influence embryologic outcomes, including reduced blastulation and increased aneuploidy rates.

Other studies, however, have suggested no adverse impact on fertilization rate, quality of embryos, and clinical outcomes including implantation rate when comparing IVF cycles in the presence of leukocytospermia to those cycles without leukocytospermia [2, 9, 10]. One study demonstrated that despite lower semen analysis parameters in the leukocytospermia group at the time of IVF and ICSI, there was no significant difference in the clinical pregnancy rate and live birth rate compared to the non-leukocytospermia group [11]. A recent meta-analysis of 28 case-control studies comparing the fertilization rate after ART for 254 leukocytospermic cases and 3613 non-leukocytospermic controls also found no significant difference between the two groups [2]. However, the majority of studies completed to date have been relatively small in sample size and none has evaluated the impact of leukocytospermia in preimplantation genetic testing for aneuploidy (PGT-A) cycles despite the possible influence of leukocytospermia on aneuploidy rate [8].

With the increased uptake of ART treatments, including IVF with ICSI and PGT-A among infertile couples [12], it is presumed that the extensive sperm processing involved with these cycles, including the use of single sperm for fertilization, should likely overcome the impact of leukocytospermia. Yet, as described in the aforementioned studies, clinical data thus far has been limited and conflicting. The objective of the current study was to investigate in a large sample size whether leukocytospermia affects clinical and embryologic outcomes in IVF with ICSI and PGT-A cycles.

## Materials and methods

### Study design

This was a retrospective cohort study at a single university-affiliated fertility practice. Patients undergoing IVF with ICSI and PGT-A at Reproductive Medicine Associates of New Jersey (RMANJ, Basking Ridge, NJ, USA) between January 2012 to December 2021 were included. Patients using frozen sperm, donor sperm, or surgical sperm were excluded from analysis. Patients undergoing a fresh embryo transfer as well as third-party cycles, including those utilizing donor eggs and/or a gestational carrier, were also excluded. Given that spermatogenesis takes approximately 90 days [13], the presence of leukocytospermia was evaluated in the semen analysis closest to oocyte retrieval and within 3 months of IVF treatment. Institutional review board approval was obtained to evaluate the retrospective data (Advarra IRB, Pro00027158).

### Semen analysis and evaluation for leukocytospermia

Semen analysis was performed according to WHO criteria [1]. Sperm samples were collected via ejaculation and obtained in sterile, labeled containers after 2 to 5 days of abstinence. After liquefaction, semen was evaluated for volume, viscosity, pH, sperm concentration, progressive motility, and morphology.

Leukocytospermia was diagnosed via peroxidase staining. A solution of Leucoscreen stain and hydrogen peroxide was combined with the semen specimen on a slide. After a minimum of 2 min, a total of 100 sperm were counted and the number of white blood cells (WBCs) stained brown due to the peroxidase reaction within the same fields were counted. The WBC concentration was then determined by dividing the number of WBCs by the number of sperm counted and multiplying by the sperm concentration. A concentration greater than 1 × 10^6^ white blood cells/mL was indicative of leukocytospermia [1, 14].

### Ovarian stimulation, IVF with ICSI, and embryo transfer

Patients underwent controlled ovarian hyperstimulation, most commonly using a gonadotropin-releasing hormone (GnRH) antagonist protocol. Decisions regarding the IVF stimulation protocol, dosing of medications, and administration of trigger (GnRH agonist and/or hCG) were at the discretion of the patient’s physician, following standard clinic practice. Oocyte retrieval via ultrasound-guided aspiration was performed 36 h after triggering final oocyte maturation. On the day of oocyte retrieval, fresh ejaculate sperm was evaluated for volume, concentration, progressive motility, and morphology. Density gradient centrifugation or simple wash was performed depending on the specimen quality. The majority of cases (> 80%) were processed via density gradient centrifugation with simple wash performed in specific circumstances (e.g., severe oligozoospermia). ICSI was performed on all mature (metaphase II) oocytes. The processed sperm was placed in polyvinylpyrrolidone droplets prior to microinjection to allow for selection and immobilization. The ICSI pipette was lowered onto the sperm’s tail until the sperm was immotile with a kink in the tail. The sperm were then aspirated, tail first, into the ICSI micropipette. The micropipette was then moved to the microdrop containing the denuded MII oocyte. The oocyte was held in position with the holding pipette, ensuring the polar body was at the 12 or 6 o’clock position to avoid damage to the meiotic spindle. The injection needle, containing the immobilized sperm near the tip, was then introduced along the *x*-axis of the oocyte at the 3 o’clock position through the zona pellucida and into the oolemma towards the 9 o’clock position. Once the oolemma was broken, the sperm was released into the oocyte with minimal surrounding media and the needle was withdrawn. This process was repeated for each MII oocyte [15]. Laser-assisted hatching of the zona pellucida was routinely performed on day 3 of embryo development. A 15–20-μm hole using an infrared 1.48-μm diode laser was made as per standard protocol [15]. Embryos were cultured through the expanded blastocyst stage and PGT-A via trophectoderm biopsy was performed [16]. Embryos were subsequently vitrified on days 5, 6, or 7 if they were of grade 4CC or higher based on the modified Gardner scoring system as per clinic protocol [17, 18]. The blastocyst was evaluated based on rate of expansion (1–6), development of the inner cell mass (A, B, C), and the trophectoderm (A, B, C). For example, based on this scoring system, an expansion grade 4 was consistent with an expanded blastocyst (blastocoel volume larger than the blastocyst and thinning of the zona pellucida). Inner cell mass grade C was consistent with very few cells, and trophectoderm grade C was consistent with very few large cells. Euploid embryo transfer was performed in a subsequent cycle after obtaining adequate endometrial proliferation of at least 7 mm in thickness. The most common endometrial preparation protocol was a programmed cycle using oral estrogen followed by intramuscular injections of progesterone in oil. However, other protocols, including a modified natural cycle protocol with hCG trigger followed by vaginal progesterone support as well as gonadotropin-stimulated cycles with hCG trigger followed by progesterone support, were also utilized at the discretion of the patient’s physician. Patients continued with progesterone supplementation until 10 weeks gestational age.

### Outcome measures

The primary outcome of our study was live birth rate (LBR). Secondary clinical outcomes included clinical pregnancy rate (CPR), defined as the presence of an intrauterine gestational sac and yolk sac, clinical loss rate (CLR), defined as a loss after a confirmed intrauterine pregnancy, and sustained implantation rate (SIR), defined as the presence of an ongoing pregnancy beyond 8 weeks gestational age. Secondary embryologic outcomes included fertilization rate, defined as the presence of two pronuclei (2PN) per mature oocyte (M2) as well as blastulation rate (calculated per 2PN) and aneuploidy rate.

### Statistical analysis

Mean, standard deviation, and 95% confidence intervals were used to describe descriptive data. Pearson’s chi-square test was used for comparison of categorical variables. *T*-test was used for comparison of continuous variables. A generalized estimation equation with multivariable regression to account for multiple cycles in the same patient, year of treatment, and to control for differences in baseline characteristics, including age (male and female), and semen analysis parameters (volume, concentration, total motile count) was performed. *p* < 0.05 was considered statistically significant. Statistical analyses were conducted using SPSS statistical software Version 27.0 (Armonk, NY: IBM Corp.).

## Results

A total of 5435 IVF cycles were included for analysis. The overall prevalence of leukocytospermia within 3 months prior to IVF was 33.9% (*n* = 1843). Baseline characteristics including patient’s age, antimullerian hormone (AMH), day 3 follicle stimulating hormone (D3 FSH), and body mass index (BMI) were not statistically different when comparing cycles with leukocytospermia to those without leukocytospermia (Table 1). Male partner’s age was significantly higher in the group with leukocytospermia, 37.3 years old vs. 36.9 years old, *p* = 0.004. There were significant differences in semen analysis parameters when comparing cycles with leukocytospermia to those without leukocytospermia. The total motile sperm count (million per ejaculate) was higher in the leukocytospermia group compared to the group without leukocytospermia (84.1 vs 78.7, *p* = 0.023). The sperm concentration (million per mL) was also greater in the leukocytospermia group (63.1 vs. 54.6, *p* <0.01).

**Table 1:** Baseline patient characteristics

Characteristic	Leukocytospermia present, ***N*** = 1843	Leukocytospermia absent, ***N*** = 3592	***p*** value
Age in years	35.3 ± 3.9	35.1 ± 3.8	0.091
AMH (ng/mL)	3.8 ± 4.3	4.0 ± 4.4	0.249
D3 FSH (IU/L)	7.9 ± 2.8	7.9 ± 2.9	0.682
BMI (kg/m^2^)	25.9 ± 5.6	25.9 ± 5.4	0.746
Partner’s age in years	37.3 ± 5.3	36.9 ± 5.0	0.004
Semen analysis parameters			
Total motile count (million per ejaculate, mean (95% CI)	84.1 (80.3–87.9)	78.7 (76.1–81.4)	0.023
Volume (mL), mean (95% CI)	2.5 (2.3–2.5)	2.7 (2.6–2.7)	0.001
Concentration (million per mL), mean (95% CI)	63.1 (61.1–65.0)	54.6 (53.3–55.9)	< 0.01

Data presented as mean ± standard deviation, unless stated otherwise

**Fig. 1:**
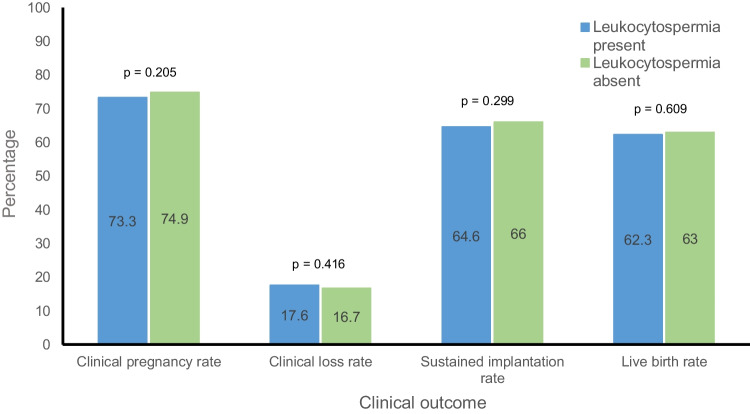
Clinical outcomes after first euploid embryo transfer

**Fig. 2:**
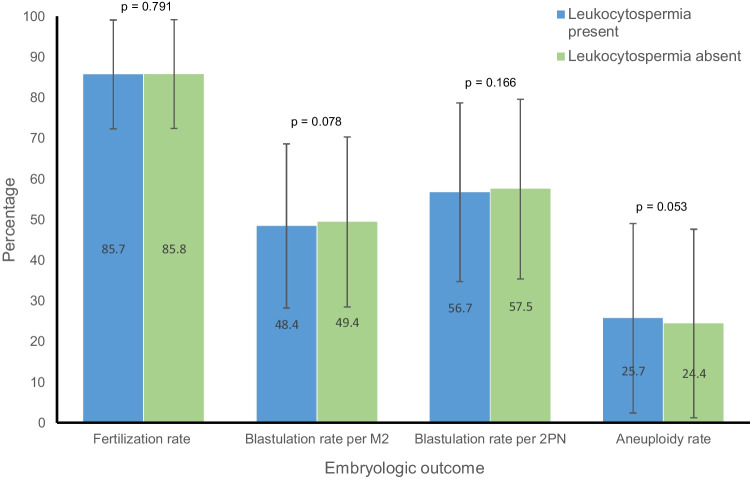
Embryologic outcomes after first euploid embryo transfer

The live birth rate after the first euploid embryo transfer did not significantly differ between the two groups (62.3% vs. 63%, *p* = 0.625). As described in Fig. 1, the CPR after the first euploid embryo transfer was similar between the two groups (73.3% vs 74.9%, *p* = 0.213), as was the CLR (17.6% vs 16.7%, *p* = 0.423) and the SIR (64.6% vs. 66%, *p* = 0.305). Figure 2 describes embryologic outcomes in detail, stratified by the presence or absence of leukocytospermia. The fertilization rate (2PN) was similar in those with and without leukocytospermia (85.7% vs. 85.8%, *p* = 0.791) as was the blastulation rate per 2PN (56.7% vs. 57.5%, *p* = 0.166). The aneuploidy rate was slightly higher in cycles with leukocytospermia, but this did not reach statistical significance (25.7% vs 24.4%, *p* = 0.053).

To account for multiple cycles completed in the same patient as well as year of treatment, a generalized estimation equation was employed with multivariable logistic regression to control for male partner’s age, female patient’s age, and semen analysis parameters. This demonstrated that the presence of leukocytospermia did not adversely influence any clinical outcomes. The live birth rate was not significantly different between the two groups (adjusted OR 0.878; 95% CI, 0.680–1.138; *p* = 0.325). The clinical pregnancy rate was similar between the groups (adjusted OR 0.876; 95% CI, 0.663–1.157; *p* = 0.351) and the sustained implantation rate also did not differ between the two groups (adjusted OR 0.838; 95% CI, 0.650–1.080; *p* = 0.172). The presence of leukocytospermia was also evaluated as a continuous linear variable to determine if the quantity (severity) of leukocytospermia impacted outcomes. The live birth rate was not significantly different from those patients with a greater quantity of leukocytospermia (adjusted OR 1.030; 95% CI, 0.986–1.076; *p* = 0.184). A ROC analysis also confirmed that the severity of leukocytospemria was not predictive of live birth outcome (AUC = 0.496; 95% CI, 0.480–0.513).

## Discussion

To date, the clinical significance of leukocytospermia and its effect on IVF and ICSI outcomes has been equivocal. Our study demonstrates that the presence of leukocytospermia just prior to IVF and ICSI does not adversely impact clinical or embryologic outcomes in comparison to cycles without leukocytospermia. In our study, the paternal age and semen analysis parameters, including TMC and sperm concentration, were greater in the leukocytospermia group. While the aneuploidy rate was slightly greater in the leukocytospermia group, it did not reach significance. Even after adjusting for potential confounding factors, the results persisted with no significant differences in outcomes seen between the two groups.

Our findings are consistent with several prior studies which have similarly demonstrated no adverse impact of leukocytospermia on clinical outcomes and fertilization rates [2, 9]. While some studies have shown reduced semen analysis parameters and negative clinical outcomes in the presence of leukocytospermia [3–8, 11], our results did not demonstrate this. The potential impact of various sperm processing techniques, sample sizes, and patient etiologies may account for the differing results seen in prior studies. Regardless, it appears that IVF and ICSI likely overcome any concerns with semen analysis parameters. The extensive sperm preparation methods used in IVF, including density-gradient centrifugation, likely improve the concentration and quality of spermatozoa, and moreover, the use of ICSI can further overcome many concerns regarding semen parameters.

The question of whether threshold for pathologic leukocytospermia remains to be elucidated. It has been shown that the presence of some WBCs in the semen is likely beneficial for immunosurveillance, quality control, and removal of abnormal sperm cells, thereby facilitating successful fertilization [19, 20]. Small amounts of ROS released in the presence of leukocytospermia may also be important for critical steps involved in reproduction including capacitation, acrosomal reaction, and sperm-oocyte fusion [19]. Our study findings suggest that in IVF with ICSI cycles, there may not be a pathological threshold beyond which adverse outcomes may occur as the embryologic and clinical outcomes were similar across all patients even when stratifying based on severity of leukocytospermia. However, additional studies are needed, stratifying outcomes based on severity of leukocytospermia to determine if there is a particular threshold above which leukocytospermia may result in adverse outcomes. Furthermore, it would be valuable to evaluate the prevalence of leukocytospermia among the fertile male population in comparison to the infertile population to determine if its presence is truly pathologic or rather ubiquitous, especially given its prevalence among asymptomatic male infertility patients.

A semen analysis including testing for leukocytospermia is routinely performed to identify factors that may contribute to male factor infertility. However, the ability of semen parameters on a standard semen analysis to predict ART outcomes is poor [21]. Given the lack of association between leukocytospermia and adverse outcomes in IVF with ICSI and PGT-A cycles seen in our study, evaluating for its presence in this population may not be necessary prior to treatment (if the patient is otherwise asymptomatic with no evidence of infection/inflammation). However, since leukocytospermia has been linked to increased DNA fragmentation, directly testing for DNA fragmentation prior to IVF treatment may be more helpful. Several studies have suggested a link between higher sperm DNA fragmentation and blastocyst aneuploidy rate [22, 23]. In our study, the aneuploidy rate was slightly higher in the leukocytospermia group but did not reach significance. Additional prospective studies are needed to further evaluate this.

There are important limitations to our study. This study evaluated outcomes among patients undergoing IVF with ICSI and PGT-A. The potential adverse impact of leukocytospermia may have been overcome by the use of ICSI and/or PGT-A. Thus, the results of the current study may not be generalizable to patients undergoing conventional IVF without PGT-A testing. Additional studies with these populations are required to further understand the impact of leukocytospermia on IVF outcomes. Furthermore, while peroxidase staining is the standard method for detecting leukocytospermia given that peroxidase-positive granulocytes are the predominant form of leukocytes in semen [1, 24, 25], more precise techniques exist to identify seminal fluid white blood cells including immunocytochemical staining using antibodies and flow cytometry [26]. However, a prior study evaluating the presence of leukocytospermia using the more accurate but time-consuming and expensive flow cytometry technique also showed no significant negative effects on IVF and ICSI outcomes [9]. Another important limitation in our study was that we were not able to distinguish which patients among those with leukocytospermia had an active genital tract inflammatory process or infection (e.g., sexually transmitted infection, testicular injury, prostatitis, urethritis). Stratifying based on symptomatic vs. asymptomatic leukocytospermia in future studies would be beneficial to further understand the impact of leukocytospermia on outcomes. However, the ability of a leukocytospermia screen to identify patients with a potential underlying infectious etiology has recently come into question in itself. A recent study has demonstrated that leukocytospermia is not an informative predictor of positive semen culture in infertile men [27]. No significant difference in the proportion of men with leukocytospermia was found between men with positive and negative semen cultures (11/54 (21%) vs 120/469 (26%)) and relying on a positive leukocytospermia test to then proceed with a semen culture missed 80% of positive semen cultures [27]. Finally, we were not able to capture the impact of antibiotic and/or anti-inflammatory treatment on leukocytospermia in our study population given the heterogeneity regarding when to treat, duration of treatment, and antibiotic choice [28, 29]. There remains no consensus across urology nor reproductive science societies regarding management of leukocytospermia with varying recommendations across guidelines [29]. Future studies should attempt to evaluate the impact of treatment regimens on outcomes. However, the results in our study suggest that antibiotic treatment, in particular for asymptomatic male patients prior to IVF with ICSI and PGT-A, is likely not warranted.

## Conclusion

Our study is the largest study to date to demonstrate that the presence of leukocytospermia (≥ 1 million WBC/mL semen) within 3 months prior to IVF with ICSI and PGT-A does not adversely impact clinical outcomes including live birth rate, clinical pregnancy rate, and sustained implantation rate. Leukocytospermia also does not appear to negatively impact fertilization, blastulation, or aneuploidy rates.

## References

[1] World Health Organization H (2021). WHO laboratory manual for the examination and processing of human semen.

[2] Castellini C (2020). Relationship between leukocytospermia, reproductive potential after assisted reproductive technology, and sperm parameters: a systematic review and meta-analysis of case-control studies. Andrology.

[3] Sharma R, et al. Relevance of leukocytospermia and semen culture and its true place in diagnosing and treating male infertility. World J Mens Health. 2022;40(2):191–207. 10.5534/wjmh.210063 PMC8987138 34169683

[4] Yilmaz S (2005). Effects of leucocytospermia on semen parameters and outcomes of intracytoplasmic sperm injection. Int J Androl.

[5] Agarwal A (2014). Reactive oxygen species and sperm DNA damage in infertile men presenting with low level leukocytospermia. Reprod Biol Endocrinol.

[6] Das S (2022). Bacteriospermia and male infertility: role of oxidative stress. Adv Exp Med Biol.

[7] Aitken RJ (1995). Analysis of sperm movement in relation to the oxidative stress created by leukocytes in washed sperm preparations and seminal plasma. Hum Reprod.

[8] Derbel R (2021). Relationship between nuclear DNA fragmentation, mitochondrial DNA damage and standard sperm parameters in spermatozoa of infertile patients with leukocytospermia. J Gynecol Obstet Hum Reprod.

[9] Ricci G (2015). Effect of seminal leukocytes on in vitro fertilization and intracytoplasmic sperm injection outcomes. Fertil Steril.

[10] Cavagna M (2012). The influence of leukocytospermia on the outcomes of assisted reproductive technology. Reprod Biol Endocrinol.

[11] Qiao X (2022). Effects of leukocytospermia on the outcomes of assisted reproductive technology. Andrologia.

[12] Bosch E, De Vos M, Humaidan P (2020). The future of cryopreservation in assisted reproductive technologies. Front Endocrinol (Lausanne).

[13] Heller CG, Clermont Y (1963). Spermatogenesis in man: an estimate of its duration. Science.

[14] WH Organization (2010). WHO laboratory manual for the examination and processing of human semen.

[15] Rosen M, et al. Chapter 32 - gamete and embryo manipulation. In: Strauss JF, Barbieri RL, editors. Yen & Jaffe’s reproductive endocrinology, physiology, pathophysiology and clinical management. 8th ed. Elsevier; 2019. pp. 823–856.

[16] Tiegs AW (2021). A multicenter, prospective, blinded, nonselection study evaluating the predictive value of an aneuploid diagnosis using a targeted next-generation sequencing-based preimplantation genetic testing for aneuploidy assay and impact of biopsy. Fertil Steril.

[17] GDS W, Janson R (1999). In vitro culture of human blastocyst. Towards reproductive certainty: infertility and genetics beyond 1999.

[18] Niederberger C (2018). Forty years of IVF. Fertil Steril.

[19] Gill K, et al. Male infertility coexists with decreased sperm genomic integrity and oxidative stress in semen irrespective of leukocytospermia. Antioxidants (Basel). 2022;5;11(10):1987. 10.3390/antiox11101987 PMC9598546 36290709

[20] Barraud-Lange V (2011). Seminal leukocytes are good samaritans for spermatozoa. Fertil Steril.

[21] Schlegel PN (2023). Can sperm quality affect reproductive outcomes?. Fertility and Sterility.

[22] Fu W (2023). High sperm DNA fragmentation increased embryo aneuploidy rate in patients undergoing preimplantation genetic testing. Reprod Biomed Online.

[23] Yang H (2022). Correlation study of male semen parameters and embryo aneuploidy in preimplantation genetic testing for aneuploidy. Front Endocrinol (Lausanne).

[24] Johanisson E (2000). *Evaluation of* ‘*round cells*’ *in semen analysis: a comparative study*. Hum Reprod Update.

[25] Wolff H (1995). The biologic significance of white blood cells in semen. Fertil Steril.

[26] Schlegel PN (2021). Diagnosis and treatment of infertility in men: AUA/ASRM guideline part I. Fertil Steril.

[27] Ventimiglia E (2020). Leukocytospermia is not an informative predictor of positive semen culture in infertile men: results from a validation study of available guidelines. Hum Reprod Open..

[28] Brunner RJ, Demeter JH, Sindhwani P (2019). Review of guidelines for the evaluation and treatment of leukocytospermia in male infertility. World J Mens Health.

[29] Velez D, Ohlander S, Niederberger C (2021). Pyospermia: background and controversies. F S Rep.

